# Effect of dietary supplementation with *Lactobacillus acidophilus* D2/CSL (CECT 4529) on caecum microbioma and productive performance in broiler chickens

**DOI:** 10.1371/journal.pone.0176309

**Published:** 2017-05-04

**Authors:** Alessandra De Cesare, Federico Sirri, Gerardo Manfreda, Paola Moniaci, Alberto Giardini, Marco Zampiga, Adele Meluzzi

**Affiliations:** 1 Department of Agricultural and Food Sciences, *Alma Mater Studiorum* - University of Bologna, Ozzano dell’Emilia, Bologna, Italy; 2 Centro Sperimentale del Latte, Zelo Buon Persico, Lodi, Italy; Leibniz-Institut fur Pflanzengenetik und Kulturpflanzenforschung Gatersleben, GERMANY

## Abstract

This study examines the effects of the dietary supplementation with *Lactobacillus acidophilus* D2/CSL (CECT 4529) (LA) on productive performances, incidence of foot pad dermatitis and caecum microbioma in broiler chickens. A total of 1,100 one-day old male Ross 308 chicks were divided into 2 groups of 16 replicates with 25 birds each and reared from 1–41 d. One group was fed a basal diet (CON) and the other group the same diet supplemented with LA. Caecum contents were collected from 4 selected birds at day one and 5 selected birds at the end of the rearing period. Then, they were submitted to DNA extraction and whole DNA shotgun metagenomic sequencing. Overall, the LA supplementation produced a significant beneficial effect on body weight gain between 15–28 d and improved feed conversion rate in the overall period. On the contrary, litter moisture, pH and incidence of the foot pad lesions were not affected by LA. Birds treated with LA showed a lower occurrence of pasty vent at both 14 and 28 d. At the end of the rearing period, *Lachanospiraceae* were significantly higher in LA birds in comparison to CON (17.07 vs 14.39%; P = 0.036). Moreover, *Ruminococcus obeum*, *Clostridium clostridioforme*, *Roseburia intestinalis*, *Lachnospiraceae bacterium* 14-2T and *Coprococcus eutactus* were significantly higher in LA birds in comparison to CON. The relative abundance of *Lactobacillus acidophilus* was comparable between LA and CON groups. However, a positive effect was observed in relation to the metabolic functions in the treated group, with particular reference to the higher abundance of β-glucosidase. In conclusion, the LA supplementation improved broiler productive performances and metabolic functions promoting animal health.

## Introduction

The intestinal microbiota of homoeothermic animals constitutes a complex ecosystem composed by a large variety of microorganisms. It plays an important role in maintaining the host normal gut functions and health, and its imbalance, or dysbiosis, can produce negative effects on gut physiology [1]. Clinical signs of dysbiosis in broilers are thinning of the small intestine, increased water content and presence of indigested residues in the faces [2]. Autochthonous *Lactobacillus* species, such as *L*. *acidophilus*, can be identified in the gastro-intestinal (GI) tract of broilers raised under commercial conditions. *Lactobacilli* become established in the chicken GI tract soon after hatching, and their metabolic activity lowers the digesta pH, which, in turn, inhibits the proliferation of enterobacteria and other unwanted bacteria [3, 4]. However, the microbiota composition changes with ageing until a labile homeostasis is reached [5–7]. Furthermore, due to intensive rearing systems, farm animals are very susceptible to enteric dysbiosis [8].

Probiotics, or direct-fed microbials, have been defined as “live microorganisms that, when administered in adequate amounts, confer a health benefit on the host” [9]. Modes of action of probiotic Lactic Acid Bacteria (LAB) that have been proposed include: competitive exclusion toward harmful bacteria, alteration of microbial and host metabolism, stimulation of immunity [2, 6, 8, 10–12]. *Lactobacillus* strains have been described as beneficial additives because of their effects in promoting poultry production performance [12–14]. Some authors highlight the role of probiotics as a sound alternative to antibiotic growth promoters [2, 15, 16]. However, kind of probiotic strain [17], dosage (i.e., colony forming unit (cfu)/bird/day), which should be modulated according to the flock health status and/or the farm hygienic conditions, as well as treatment duration, are among the critical factors influencing a probiotic efficacy. Other important variables are probiotic conservation and distribution technology, feed composition, also in terms of presence of antimicrobial agents and probiotic carriers (i.e., feed or drinking water) [18].

In the past researchers investigated the impact of the administration of probiotics on broiler GI tract by testing those microorganisms that could be recovered on growth media. However, they represent less that 20% of bacterial taxa inhabiting the poultry GI tract [19]. Within the last decade, the development of high-throughput sequencing technologies, targeting the whole set of genes within a system, gained a relatively unbiased view of both GI community structure (i.e., bacterial species richness and distribution) and functional (metabolic) potential [20].

The aim of this study was to evaluate the effects of the supplementation with *Lactobacillus acidophilus* D2/CSL (CECT 4529) in broiler chicken diets on productive performances, foot pad dermatitis and caecum microbioma, in terms of bacteria population and metabolic functions, by whole DNA shotgun metagenomic sequencing.

## Materials and methods

### Animals and treatments

The experiment was approved by the Ethical Committee of the University of Bologna on 17/3/2014 (ID: 10/79/2014). A total of 1,100 one-day old male Ross 308 chicks, obtained from the same breeder flock and hatching session, were used. Birds were vaccinated against infectious bronchitis virus, Marek’s disease virus, Newcastle and Gumboro diseases and coccidiosis at the hatchery. Before housing, chicks were individually weighed and divided in the following 5 classes according to their live weight: <42 g, 42–44 g, 45–47 g, 48–50 g, >50 g. The first and the last groups were discarded, while the remaining were distributed in 32 pens (2.5 m^2^ each) at the stocking density of 10 chicks/m^2^ (25 birds/pen), while maintaining the same class distribution of live-weight of the population. Pens were equipped with pan feeders, to assure at least 2 cm/bird of front space, and an independent drinking system with 1 nipple/5 birds. Feeders were of identical manufacture, type, size, color, and other notable physical features. Each pen was equipped with an individual bin, clearly labeled as reservoir for the experimental feeds. On a daily basis, the experimental feeds were manually transferred from the bin to the feeder. Any change in the diet was made uniformly for all animals. Feed and water were provided for *ad libitum* consumption. At each diet switch, feeders were emptied, orts were weighed back and the feeders were filled with the diets described below. Twice daily observations were recorded for general flock condition, temperature, lighting, water, feed, litter condition and mortality. The experiment lasted 41 days when birds reached the slaughter weight of about 2.8 kg of live weight. Photoperiod and temperature programs were set up according to the European welfare regulation 43/2007 [21].

The chicks were divided into 2 groups of 16 replicates with 25 birds each, fed with the basal diet (control group, CON) (Table 1) or the basal diet supplemented with *L*. *acidophilus* D2/CSL (bacterial concentration of 5.0 x 10^10^ cfu g^–1^) at the dosage of 20 g ton^-1^ feed (LA group). The probiotic strain *L*. *acidophilus* D2/CSL has been isolated from the GI tract of a healthy adult chicken [22] and supplied by Centro Sperimentale del Latte S.r.l. (Lodi, Italy).

**Table 1:** Basal diets composition.

	Starter (0–14 d)	Grower (15–28 d)	Finisher (29–41 d)
**Ingredients, g/100 g**			
Corn	42.17	34.96	12.73
White corn	0.00	0.00	15.00
Wheat	10.00	20.00	25.01
Sorghum	0.00	0.00	5.00
Soybean meal	23.11	20.63	17.60
Expanded soybean	10.00	10.00	13.00
Sunflower	3.00	3.00	3.00
Corn gluten meal	4.00	3.00	0.00
Soybean oil	3.08	4.43	5.48
Dicalcium phosphate	1.52	1.20	0.57
Calcium carbonate	0.91	0.65	0.52
Sodium bicarbonate	0.15	0.10	0.15
Salt	0.27	0.27	0.25
Choline chloride	0.10	0.10	0.10
Lysine sulphate	0.59	0.55	0.46
Dl-methionine	0.27	0.29	0.30
Threonine	0.15	0.14	0.14
Xylanase	0.08	0.08	0.08
Phytase	0.10	0.10	0.10
Vitamin-mineral premix^1^	0.50	0.50	0.50
**Proximate composition, g/100g**			
Dry matter	88.57	88.65	88.64
Protein	22.70	21.49	19.74
Lipid	7.06	8.24	9.74
Fiber	3.08	3.04	3.07
Ash	5.85	5.17	4.49
Lys	1.38	1.29	1.21
Ca	0.91	0.80	0.59
P	0.63	0.57	0.46
ME (kcal/kg)	3,076	3,168	3,264

^1^ Provided the following per kg of diet: vitamin A (retinyl acetate), 13,000 IU; vitamin D3 (cholecalciferol), 4,000 IU; vitamin E (DL-α_tocopheryl acetate), 80 IU; vitamin K (menadione sodium bisulfite), 3 mg; riboflavin, 6.0 mg; pantothenic acid, 6.0 mg; niacin, 20 mg; pyridoxine, 2 mg; folic acid, 0.5 mg; biotin, 0.10 mg; thiamine, 2.5 mg; vitamin B_12_ 20 μg; Mn, 100 mg; Zn, 85 mg; Fe, 30 mg; Cu, 10 mg; I, 1.5 mg; Se, 0.2 mg; ethoxyquin, 100 mg.

The experimental diets were weekly produced by adding the LA to the common basal diet. The feeding program included three feeding phases: Starter (0–14 d), Grower (15–28 d) and Finisher (29–41 d). The basal diet composition is given in Table 1.

### Productive performance and slaughtering traits

**Fig 1:**
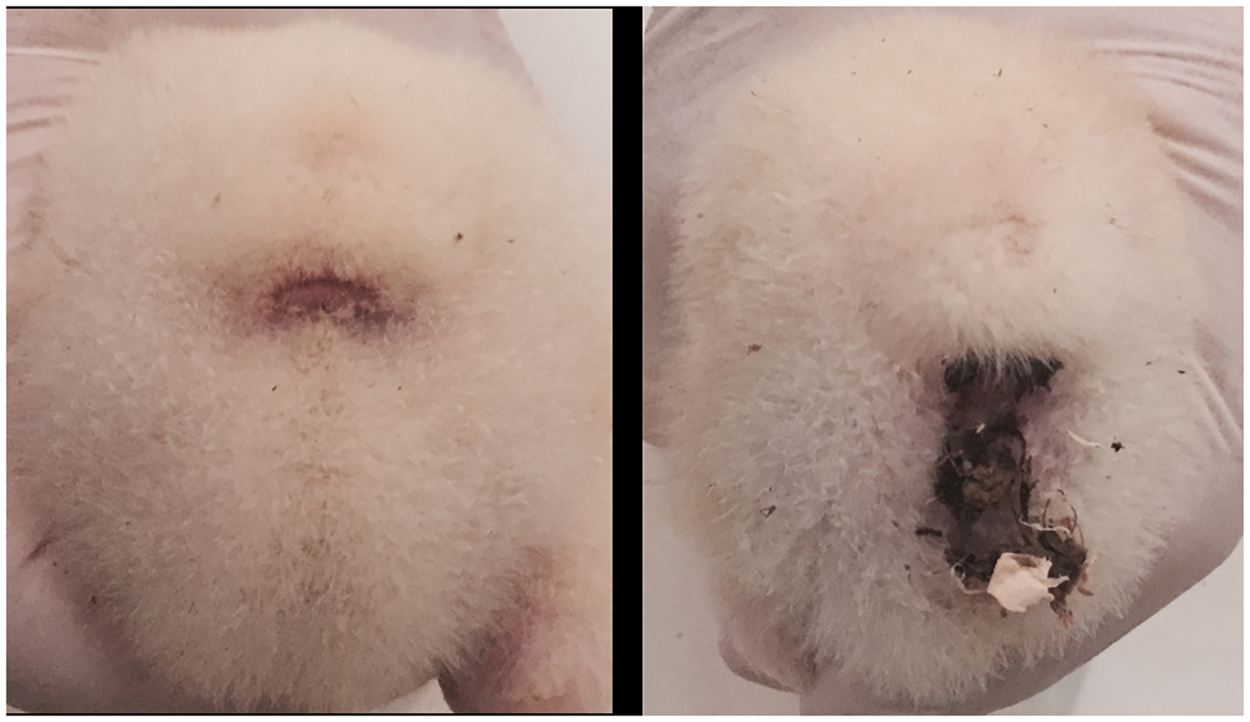
Bird showing normal vent (on the left) or pasty vent (on the right).

At housing, chicks individually weighed as previously described, in order to maintain the same class distribution of live-weight of the population within each pen, were counted and weighed on a pen basis, representing the experimental unit for productive performance measurements. Chickens and feed were weighed pen wise at 14, 28 and 41 days. Daily weight gain (DWG), feed intake (FI) and feed conversion ratio (FCR) were calculated for each feeding phase. Mortality was recorded and the above mentioned measures were adjusted for mortality. Litter moisture and pH were determined at the end of the experiment on a representative sample collected in two different areas within each pen, away from feeders and drinkers [23]. The incidence of pasty vent on a bird basis was recorded at 14 and 28 days by visual examination, based on presence or absence of sticky feces in the vent area (Fig 1).

All birds were processed at a commercial slaughterhouse and carcass yields were measured (breast, wings and legs) according to the standard procedures. Prior slaughter, the birds were subjected to a total feed withdrawal of 12 h, including the holding time of 2 h at the processing plant and processed under commercial conditions using electrical stunning (120 V, 200 Hz). Carcasses were obtained by removing head, neck, shanks and abdominal fat from bled, plucked and eviscerated birds. The incidence of foot pad dermatitis was assessed on all the birds, by collecting one foot/bird, and evaluated on a replicate basis. The feet were macroscopically examined and scored according to the available classification [24] in three classes of foot pad dermatitis (FPD): 0 = no lesions, 1 = mild lesions, and 2 = severe lesions.

### Sample collection for metagenomics

To characterize the impact of the investigated diets on the caecum microbioma, representing both the microbial populations and the genes related to their metabolic functions, four chickens were randomly selected and humanely euthanized at day 1, before starting the dietary treatment. Moreover, five chickens were randomly selected from both CON and LA groups and humanely euthanized at 41 days. The entire gastrointestinal (GI) tract of the 14 individual selected birds was dissected out and a small sample (i.e., 0.5 to 2 g) of cecum content was collected into 15-ml sterile plastic tubes. The samples collected were then stored at -80°C until further testing.

### DNA Extraction

The DNA was extracted from each caecum content using a bead-beating procedure [25]. Briefly, 0.25 g of cecal content were suspended in 1 ml lysis buffer (500 mM NaCl, 50 mM Tris-Cl, pH 8.0, 50 mM EDTA, 4% SDS) with MagNA Lyser Green Beads (Roche, Milan, Italy) and homogenized on the MagNA Lyser (Roche) for 25 sec at 6500 rpm. The samples were then heated at 70°C for 15 min, followed by centrifugation to separate the DNA from bacterial cellular debris. This process was repeated with a second 300 μl aliquot of lysis buffer. The samples were then subjected to 10 M v/v ammonium acetate (Sigma, Milan, Italy) precipitation, followed by isopropanol (Sigma) precipitation, 70% ethanol (Carlo Erba, Milan, Italy) washing and suspension in 100 ul 1X Tris-EDTA (Sigma). All samples were treated with DNase-free RNase (Roche) and incubated overnight at 4°C, before being processed through the QIAmp^®^ DNA Stool Mini Kit (Qiagen, Milan, Italy) according to manufacturer’s directions with some modifications. DNA quantity and quality were measured on a BioSpectrometer^®^ (Eppendorf, Milan, Italy).

### Metagenomic sequencing

Total DNA from each of the 14 samples was fragmented and tagged with sequencing adapters using the Nextera XT DNA Library Preparation Kit (Illumina, San Diego, CA). Whole genome sequencing was performed using the HiScanSQ sequencer (Illumina) at 100 bp in paired-end mode. Metagenomic sequencing yielded an average of 6.841 million mapped reads/sample, with a Phread quality score always higher than 30. Following sequencing, all reads were assessed for quality parameters and the paired end merged. The MG-RAST pipeline [26] (metagenomics.anl.gov) was used to identify the relative abundances of bacterial taxa performing a BLAST similarity search for the longest cluster representative against the M5rna database, integrating SILVA [27], Greengenes [28] and RDP [29]. Moreover, the sequenced reads were assigned to functional groups using the Kyoto Encyclopedia of Genes and Genome (KEGG) database (www.genome.jp/kegg/) [30] and the percentage of abundance was calculated.

### Statistical analysis

Data regarding productive traits were analyzed using the General Lineal Model (GLM) procedure of SAS [31]. One-way ANOVA with significance level of P < 0.05 was used to test the effect of LA supplementation on performance parameters. Data, expressed as percentage, were transformed to arc-sin before the analysis to homogenize the variance. Data regarding carcass yield and incidence of meat abnormalities were analyzed using the chi square test. The results concerning the relative abundances of bacterial taxa and functional groups were compared through the White’s non-parametric t-test, using Statistical Analysis of Metagenomic profile Software v 2.0.9 (STAMP) [32].

## Results

### Productive performances

Productive performances recorded during the trial (Table 2) were consistent with those reported by the Ross 308 performance objectives. At 14 days of age birds receiving LA resulted significantly heavier than CON (464 vs 453 g, P<0.05) and consequently DWG resulted higher (29.9 vs 29.0 g/bird/d; P<0.01). LA chicks consumed more feed than CON ones (36.3 vs 35.7 g/bird/d, P = 0.06). Mortality resulted in general low and similar in both groups, whereas pasty vent incidence was higher in CON (25.7 vs 10.7%, P<0.01) (Table 2).

**Table 2:** Productive performances and incidence of pasty vent of chickens untreated (CON) and treated with *L*. *acidophilus* (LA) separated by feeding phase.

	CON	LA	SE	P-value
*n*.	*16*	*16*		
-----------------------------------*0–14 d*--------------------------------				
**Chick body weight (g)**	46.05	46.02	0.05	0.76
**Body weight (g)**	453 b	464 a	3.38	0.03
Daily weight gain (g/bird/d)*	29.0 b	29.9 a	0.25	0.03
Daily feed intake (g/bird/d)*	35.7	36.3	0.22	0.06
Feed conversion rate*	1.229	1.217	0.008	0.28
**Mortality (%)**	0.50	1.50	0.02	0.11
**Pasty vent (%)**	25.7 A	10.7 B	0.03	< 0.01
-----------------------------------*15–28 d*--------------------------------				
**Body weight (g/bird)**	1,498 b	1,531 a	9.87	0.03
Daily weight gain (g/bird/d)*	74.6	76.1	0.63	0.09
Daily feed intake (g/bird/d)*	111	112	0.74	0.39
Feed conversion rate*	1.492	1.473	0.008	0.09
**Mortality (%)**	0.75	0.50	0.02	0.66
**Pasty vent (%)**	52.2 A	37.8 B	0.04	< 0.01
-----------------------------------*29–41 d*--------------------------------				
**Body weight (g/bird)**	2,757	2,784	13.64	0.17
Daily weight gain (g/bird/d)*	96.5	95.7	0.86	0.52
Daily feed intake (g/bird/d)*	178 a	174 b	1.29	0.03
Feed conversion rate*	1.842	1.815	0.02	0.26
**Mortality (%)**	0.75	0.75	0.02	0.82
-----------------------------------*0–41 d*--------------------------------				
**Body weight (g/bird)**	2,757	2,784	13.64	0.17
Daily weight gain (g/bird/d)*	65.83	66.31	0.37	0.37
Daily feed intake (g/bird/d)*	106.2	105.3	0.46	0.30
Feed conversion rate*	1.613 a	1.588 b	0.008	0.03
**Mortality (%)**	2.00	2.75	0.03	0.25

* corrected for mortality

^a, b^: P<0.05;

^A, B^: P<0.01.

At 28 days of age, birds receiving LA weighed 33 g more than CON (1,531 vs 1,498 g, P<0.05). FCR resulted slightly, but not significantly, improved in LA birds (1.473 vs 1.492, P = 0.09). Mortality resulted similar in both groups and incidence of pasty vent was significantly lower in LA group (37.8 vs 52.2%; P<0.01) (Table 2).

At the end of trial (41 d), LA birds showed a slightly higher body weight than CON but the difference was not statistically significant (2,784 vs 2,757, P = 0.17). Daily feed intake from 29 to 41 days was significantly lower in LA birds (173 vs 178 g/bird/d; P<0.05) (Table 2).

Overall the productive performance from 0 to 41 days (Table 2) showed that LA supplementation significantly improved feed conversion rate (1.588 vs 1.613; P<0.03). Mortality presented low values in both groups ranging from 2 (CON) to 2.75% (LA).

Carcass and cut up yields at slaughter were not affected by LA supplementation (Table 3). Litter moisture and pH resulted similar in the two groups (35.4 vs 34.8% and 8.68 vs 8.63 for CON and LA, respectively; data not shown). Similarly, the incidence of foot pad lesions was not affected by the dietary treatment (Table 4).

**Table 3:** Carcass evaluation at slaughtering of chickens untreated (CON) and treated with *L*. *acidophilus* (LA).

	CON	LA
*n*.	*386*	*384*
**Eviscerated yield (%)**	70.0	70.6
Breast* **(%)**	30.3	30.7
Legs*(%)	44.4	44.6
Unseparated wings*(%)	19.3	19.2
**Chi**^**2**^, **(P-value)**	0.998	

*calculated as a percentage of eviscerated carcass weight

**Table 4:** Incidence of foot pad lesions of chickens untreated (CON) and treated with *L*. *acidophilus* (LA).

	CON	LA
*Bird scored*, *n*.	*386*	*384*
**Score 0 (no lesions) (%)**	97.1	99.5
**Score 1 (mild lesions) (%)**	2.9	0.5
**Score 2 (severe lesions) (%)**	0.0	0.0
**Chi**^**2**^, **(P-value)**	0.401	

### Metagenomic results

The metagenomes of the 14 samples included in this study are public available from MG RAST (http://metagenomics.anl.gov/linkin.cgi?project=13081). The metagenome ID mgm 4624898.3, 4625263.3, 4625261.3 and 4625265.3 refer to samples collected at day one. The mgm 4625297.3, 4625262.3, 4625269.3, 4625304.3 and 4625316.3 refer to samples collected from the control group at 41 days. The mgm 4625288.3, 4625285.3, 4625287.3, 4625273.3 and 4625272.3 refer to samples collected from the treated group at 41 days.

#### Caecum microbiota composition

The microbiota composition of one-day old chicks is summarized in Table 5. More than 95% of bacterial population was represented by Firmicutes (85.5%) and Proteobacteria (9.61%). Both these Phyla were largely represented also in the caeca of CON and LA birds at 41 days (Table 5). The relative frequency of abundance of Firmicutes in one-day old chicks was significantly lower than that observed in both groups at 41 days (P = 0.01), whereas Proteobacteria were significantly higher (P = 0.0067).

**Table 5:** Mean relative frequency of abundance (%) of phyla, classes and families of caecum bacteria in one-day old and 41-day old chickens untreated (CON) and treated with *L*. *acidophilus* (LA).

Phylum	Class	Family	One-day	CON	LA	P value CON *vs* LA	P value One-day *vs* CON	P value One-day *vs* LA
Firmicutes			85.85	93.93	92.14	0.121	0.056	0.098
	Bacilli		43.55	20.72	18.91	0.703	0.038	0.029
		Bacillaceae	1.17	1.12	0.81	0.331	0.949	0.636
		Paenibacillaceae	0.18	0.49	0.95	0.278	0.196	0.104
		Staphylococcaceae	0.93	0.22	0.18	0.616	0.392	0.369
		Enterococcaceae	3.72	0.50	0.37	0.488	0.132	0.123
		Lactobacillaceae	33.45	17.22	15.62	0.752	0.069	0.053
		Streptococcaceae	1.79	0.44	0.32	0.678	0.030	0.029
	Clostridia		41.92	70.51	70.79	0.950	0.048	0.043
		Clostridiaceae	12.28	11.89	14.30	0.070	0.939	0.697
		Eubacteriaceae	3.47	3.87	3.73	0.828	0.839	0.893
		Lachnospiraceae	13.25	14.39	17.07	0.036	0.621	0.160
		Peptococcaceae	0.18	0.60	0.43	0.306	0.110	0.281
		Peptostreptococcaceae	1.18	3.89	3.39	0.724	0.119	0.181
		Ruminococcaceae	6.80	29.53	26.27	0.423	< 0.0001	0.001
	Erysipelotrichi		0.37	2.21	1.83	0.424	0.002	0.008
	Negativicutes		0\	0.47	0.60	0.456	0.001	0.014
Proteobacteria			9.61	1.74	2.10	0.389	0.050	0.057
	Alphaproteobacteria		0.49	0.57	0.66	0.689	0.809	0.657
	Betaproteobacteria		0.18	0.10	0.10	0.993	0.683	0.692
	Deltaproteobacteria		0.18	0.27	0.48	0.213	0.666	0.249
	Gammaproteobacteria		8.74	0.77	0.84	0.873	0.075	0.076
		Enterobacteriaceae	7.63	0.62	0.70	0.851	0.069	0.070
Actinobacteria			1.60	0.92	1.43	0.397	0.466	0.850
Bacteroidetes			0.18	0.35	0.29	0.627	0.456	0.614
Tenericutes			1.42	1.18	1.47	0.612	0.447	0.924

Within Firmicutes, in one-day old chicks Bacilli was the most abundant class, followed by Clostridia. On the contrary, at 41 days, Clostridia represented the most abundant class in both LA and CON groups (70.8 and 70.5% respectively), followed by Bacilli, presenting a relative frequency of abundance of 20.7 and 18.9%, respectively. The mean relative abundances of Clostridia and Bacilli in the birds at the end of the rearing period were significantly higher (P = 0.0086) and lower (P = 0.0094) respectively, in comparison to those of one-day old chicks (Table 5). In one-day old chicks, as well as LA and CON groups, Gammaproteobacteria was the most representative class of Proteobacteria. This class was the only one significantly higher in one-day old chicks in comparison to CON and LA groups at 41 days of age (P = 0.015 and P = 0.017 respectively). Moreover, in one-day old chicks Enterobacteriaceae was the most represented family (7.63%) in comparison to the birds at 41 days, where the relative abundances of the same family were as low as 0.77 and 0.84% in CON and LA, respectively (Table 5). Within Bacilli class, the most represented family in one-day old chicks was Lactobacillaceae (33.5%), followed by Enterococcaceae (3.72%), Streptococcaceae (1.79%) and Bacillaceae (1.17%) (Table 5). This distribution was similar in CON and LA birds at 41 days, except for Bacillaceae and Paenibacillaceae, representing the second most abundant families in CON and LA birds at 41 days, respectively.

**Fig 2:**
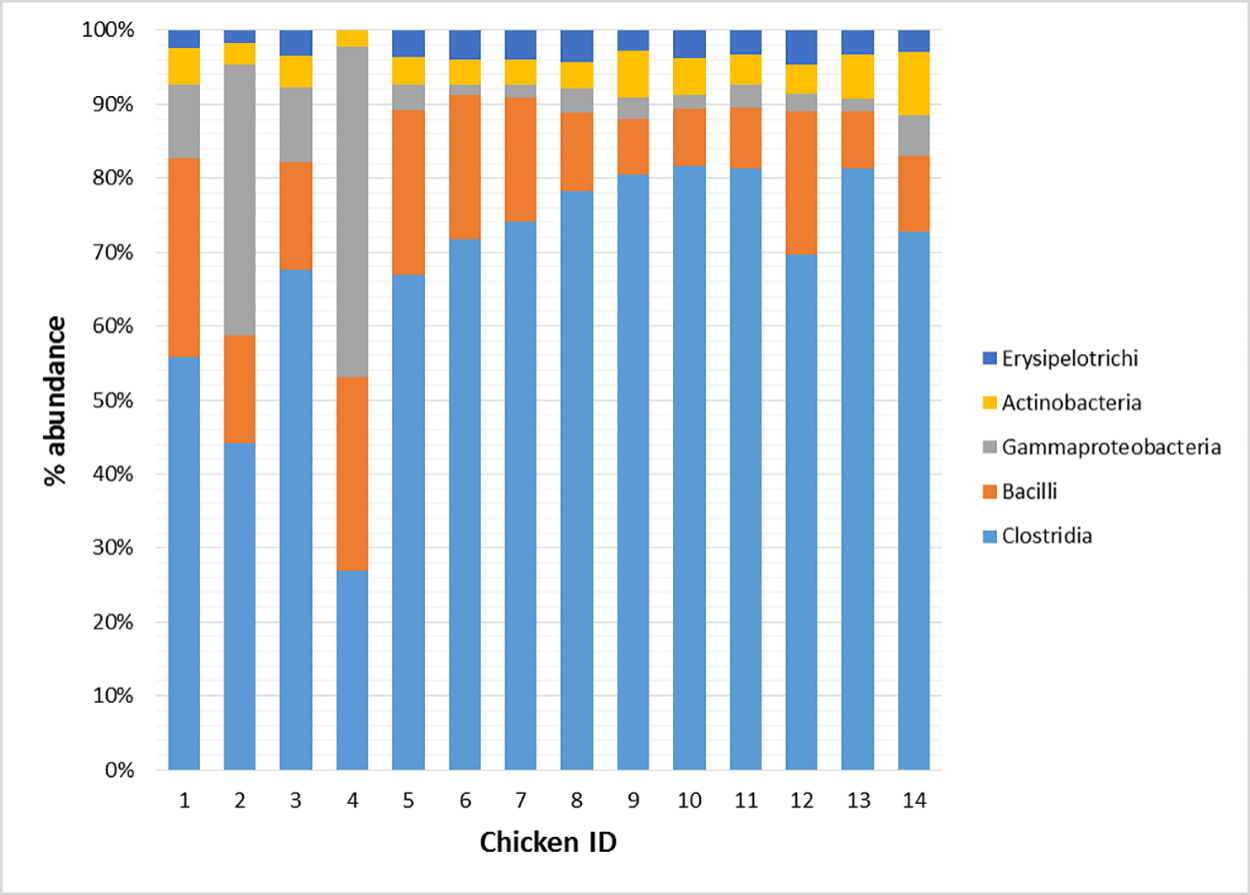
Mean relative frequency of abundance (% abundance) of most represented bacterial classes in each of the 14 chickens tested (Day 1: Chicken ID 1–4; Control 41 days: Chicken ID 5–9; Treated 41 days: Chicken ID 10–14).

Chickens identified as 1 and 3 showed a percentage of abundance of Gammaproteobacteria lower in comparison to the other one-day old chicks (i.e., 9.91 and 10.03 vs 36.47 and 44.70%) (Fig 2). The decrease of Gammaproteobacteria corresponded to a higher abundance of Clostridia (55.81 and 67.70%, respectively). On the contrary, Chicken ID 4 showed the highest abundance of Gammaproteobacteria (44.70%) and the lowest abundance of Clostridia (26.88%) in comparison with the other one-day old chicks. t 41 days the percentage of abundance of the top five classes was quite similar among chickens belonging to CON and LA groups. However, chicken ID 12 showed a higher percentage of Bacilli abundance and a lower abundance of Clostridia in comparison to the other LA chickens (Fig 2).

In comparison to one-day old chicks, Lactobacillaceae, Enterococcaceae and Streptococcaceae decreased significantly in both CON (i.e., P = 0.023, P = 0.042 and P = 0.01, respectively) and LA (i.e. P = 0.031, P = 0.013, P = 0.002, respectively) at 41 d. Lachanospiraceae was the most represented family identified within the Clostridia class in one-day old chicks (13.25%). On the contrary, in CON and LA at 41 days Ruminococcaceae was the most represented family (29.53 and 26.27%, respectively) and showed a relative frequency of abundance significantly higher than in one-day old chicks (P = 0.00044 and P = 0.0107 for CON and LA at 41 days, respectively). At the end of the rearing period Lachanospiraceae was significantly higher in LA birds in comparison to CON (17.07 vs 14.39; P = 0.036) and the same trend was observed for *Clostridiaceae* (14.30 vs 11.89%; P = 0.074) (Table 5).

**Fig 3:**
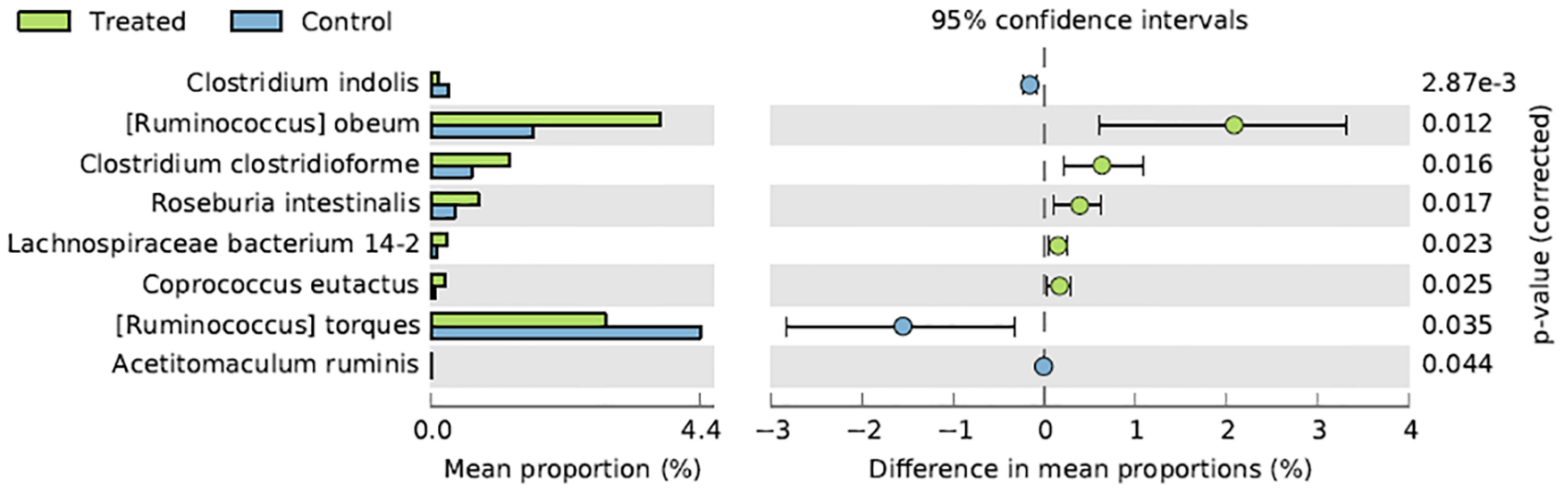
Bacterial species resulting significantly different in chickens treated with *L*. *acidophilus* (Treated) in comparison to the untreated birds (Control) at 41 days.

Overall, among the first 30 bacterial species identified in one-day old chicks the most represented species were *Lactobacillus johnsonii*, *Lactobacillus crispatus*, *Escherichia coli*, *Ruminococcus torques*, *Lactobacillus helveticus*, *Lactobacillus gasseri*, *Ruminococcus obeum*, *Ruminococcaceae bacterium* D16, *Clostrifium hylemonae* and *Eubacterium limosum* (Table 6). At 41 days, the most represented species in CON were *Faecalibacterium prausnitzii*, *Lactobacillus crispatus*, *Ruminococcus torques*, *Subdoligranulum variabile*, *Ruminococcaceae bacterium* D16, *Lactobacillus johnsonii*, *Pseudoflavonifractor capillosus*, *Ruminococcus obeum*, *Clostridium difficile* and *Blautia hydrogenotrophica*, whereas in LA they were *Faecalibacterium prausnitzii*, *Lactobacillus johnsonii*, *Ruminococcus obeum*, *Subdoligranulum variabile*, *Ruminococcus torques*, *Ruminococcaceae bacterium* D16, *Lactobacillus reuteri*, *Lactobacillus crispatus*, *Blautia hydrogenotrophica* and *Clostrium leptum* (Table 6). In relation to the bacterial species significantly different among the tested groups (Table 7), the relative frequency of abundance of *Lactobacillus crispatus* was significantly higher in one-day old chicks in comparison to LA, whereas *Ruminococcus lactaris* was significantly higher in one-day old chicks in comparison to CON. The species *Faecalibacterium prausnitzii* and *Subdoligranulum variabile* showed a significantly higher relative frequency of abundance in LA and CON birds in comparison to one-day old chicks. On the contrary, all other species were significantly higher in one-day old chicks in comparison to both LA and CON groups (Table 7). Overall, among all the bacterial species identified in CON and LA groups at 41 days, those showing a significantly higher relative frequency of abundance in LA birds were *Ruminococcus obeum*, *Clostridium clostridioforme*, *Roseburia intestinalis*, *Lachnospiraceae bacterium* 14-2T and *Coprococcus eutactus*. On the contrary, *Clostridium indolis* and *Ruminococcus torques* were significantly higher in CON (Fig 3).

**Table 6:** Mean relative frequency of abundance (%) of the 30 most representative species (MRS) of caecum bacteria in one-day old and 41-day old chickens untreated (CON) and treated with *L*. *acidophilus* (LA).

	One-day		CON		LA	
MRS^1^	Species	Mean	Species	Mean	Species	Mean
1	*Lactobacillus johnsonii*	11.36	*Faecalibacterium prausnitzii*	17.35	*Faecalibacterium prausnitzii*	14.00
2	*Lactobacillus crispatus*	6.14	*Lactobacillus crispatus*	5.62	*Lactobacillus johnsonii*	4.17
3	*Escherichia coli*	4.80	*Ruminococcus torques*	4.41	*Ruminococcus obeum*	3.76
4	*Ruminococcus torques*	3.80	*Subdoligranulum variabile*	3.26	*Subdoligranulum variabile*	2.99
5	*Lactobacillus helveticus*	2.94	*Ruminococcaceae bacterium D16*	3.10	*Ruminococcus torques*	2.86
6	*Lactobacillus gasseri*	2.73	*Lactobacillus johnsonii*	2.44	*Ruminococcaceae bacterium D16*	2.73
7	*Ruminococcus obeum*	1.98	*Pseudoflavonifractor capillosus*	2.05	*Lactobacillus reuteri*	2.44
8	*Ruminococcaceae bacterium D16*	1.98	*Ruminococcus obeum*	1.68	*Lactobacillus crispatus*	2.12
9	*Clostridium hylemonae*	1.85	*Clostridium difficile*	1.59	*Blautia hydrogenotrophica*	1.62
10	*Eubacterium limosum*	1.80	*Blautia hydrogenotrophica*	1.38	*Clostridium leptum*	1.62
11	*Clostridium bolteae*	1.74	*butyrate-producing bacterium SM4/1*	1.30	*Pseudoflavonifractor capillosus*	1.54
12	*Lactobacillus vaginalis*	1.69	*Clostridium leptum*	1.27	*Blautia sp*. *Ser8*	1.31
13	*Lactobacillus reuteri*	1.68	*Lactobacillus reuteri*	1.23	*Ruminococcus bromii*	1.29
14	*Enterococcus faecalis*	1.48	*Lactobacillus acidophilus*	1.18	*Clostridium difficile*	1.29
15	*Shigella boydii*	1.43	*Ruminococcus bromii*	1.11	*Clostridium clostridioforme*	1.29
16	*Enterococcus faecium*	1.32	*Ruminococcus albus*	1.06	*Clostridium bolteae*	1.16
17	*Clostridium asparagiforme*	1.30	*Lactobacillus vaginalis*	1.05	*butyrate-producing bacterium SM4/1*	1.03
18	*Lactobacillus delbrueckii*	1.30	*Lactobacillus helveticus*	1.02	*Ruminococcus flavefaciens*	0.94
19	*Ruminococcus lactaris*	1.18	*Ruminococcus flavefaciens*	0.90	*Lactobacillus helveticus*	0.92
20	*butyrate-producing bacterium SL7/1*	1.11	*Lactobacillus agilis*	0.87	*Lactobacillus agilis*	0.90
21	*Granulicatella adiacens*	0.93	*butyrate-producing bacterium SL7/1*	0.84	*Ruminococcus gnavus*	0.90
22	*Blautia hydrogenotrophica*	0.93	*Clostridium bartlettii*	0.84	*Clostridium scindens*	0.89
23	*Clostridium sphenoides*	0.93	*Ruminococcus gnavus*	0.83	*butyrate-producing bacterium SL7/1*	0.88
24	*Enterococcus pseudoavium*	0.92	*Anaerotruncus colihominis*	0.81	*Ruminococcus albus*	0.86
25	*Lactobacillus acidophilus*	0.87	*Butyricicoccus pullicaecorum*	0.81	*Clostridium saccharolyticum*	0.84
26	*Faecalibacterium prausnitzii*	0.87	*Clostridium bolteae*	0.80	*Lactobacillus vaginalis*	0.82
27	*Pseudoflavonifractor capillosus*	0.87	*Eubacterium hallii*	0.76	*Lactobacillus acidophilus*	0.78
28	*Lactobacillus plantarum*	0.87	*Blautia sp*. *Ser8*	0.74	*Roseburia intestinalis*	0.77
29	*Clostridium scindens*	0.80	*Dorea formicigenerans*	0.68	*Anaerotruncus colihominis*	0.70
30	*butyrate-producing bacterium SM4/1*	0.80	*Clostridium clostridioforme*	0.66	*Blautia hansenii*	0.65

^1^MRS: most represented species

**Table 7:** Statistically significant differences between means of relative frequency of abundance (%) of caecum bacterial species in one-day old and 41-day old chickens untreated (CON) and treated with *L*. *acidophilus* (LA).

	One-day	CON	LA	One-day *vs* CON	One-day *vs* LA	CON vs LA
*Species*	Mean			P-values		
*Lactobacillus johnsonii*	11.36	2.44	4.17	0.009	0.004	0.410
*Lactobacillus crispatus*	6.14	5.61	2.12	0.005	0.005	0.192
*Escherichia coli*	4.80	0.40	0.50	0.016	0.022	0.751
*Lactobacillus gasseri*	2.73	0.37	0.45	0.006	0.008	0.676
*Clostridium bolteae*	1.74	0.80	1.16	0.003	0.039	0.130
*Shigella boydii*	1.43	0.03	0.03	0.0006	0.0009	0.794
*Lactobacillus delbrueckii*	1.30	0.23	0.28	0.019	0.028	0.609
*Ruminococcus lactaris*	1.18	0.17	0.30	0.046	0.106	0.451
*Faecalibacterium prausnitzii*	0.87	17.35	14.00	< 0.0001	0.0005	0.331
*Subdoligranulum variabile*	0.62	3.26	2.99	0.002	0.003	0.665

#### Caecum metabolic genes composition

**Fig 4:**
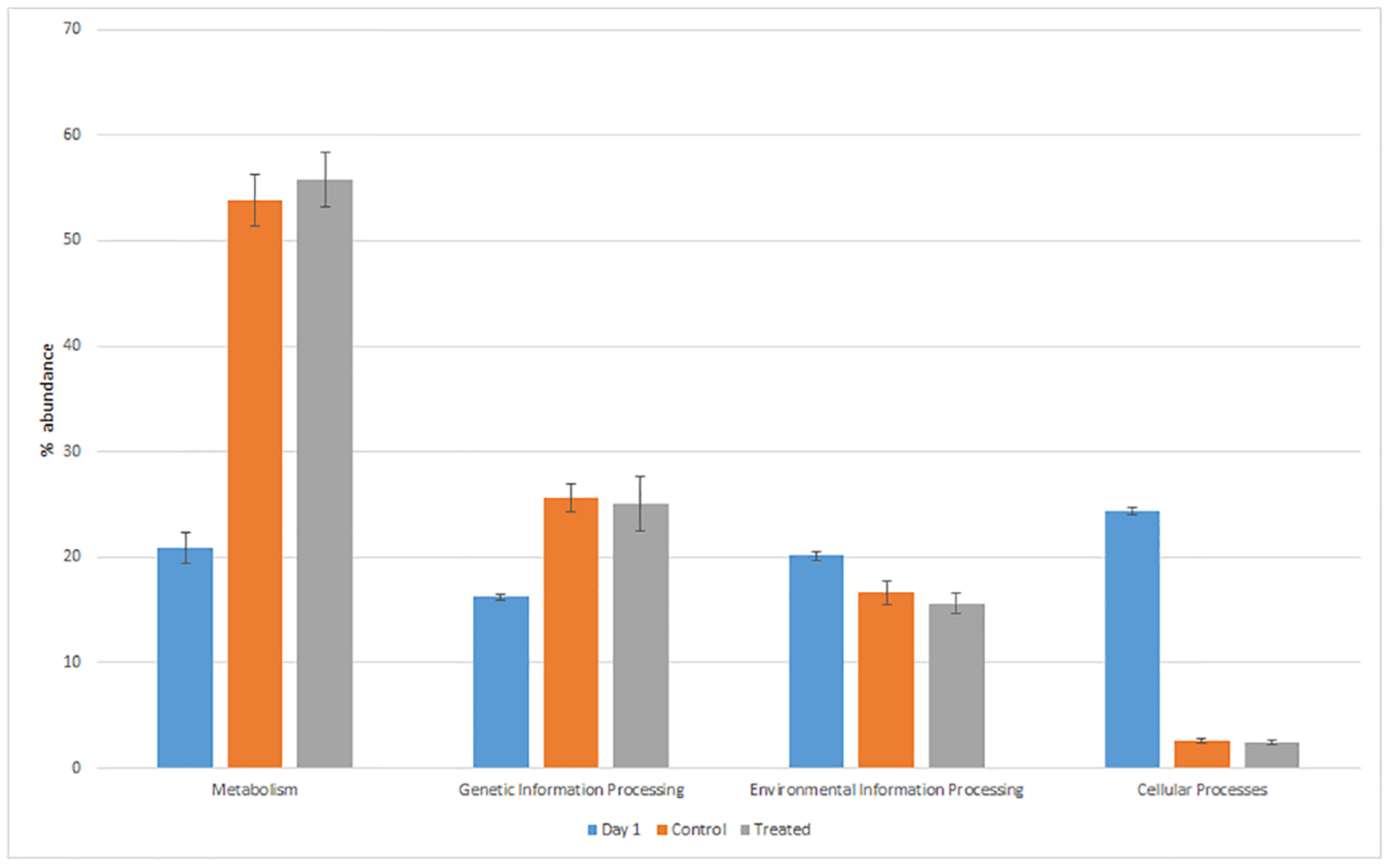
Mean relative frequency of abundance (% abundance) of the KEGG pathways in caeca of Day 1 chickens (Day 1) and in ceca of chickens treated with *L*. *acidophilus* (Treated) in comparison to the untreated birds (Control) at 41 days.

**Fig 5:**
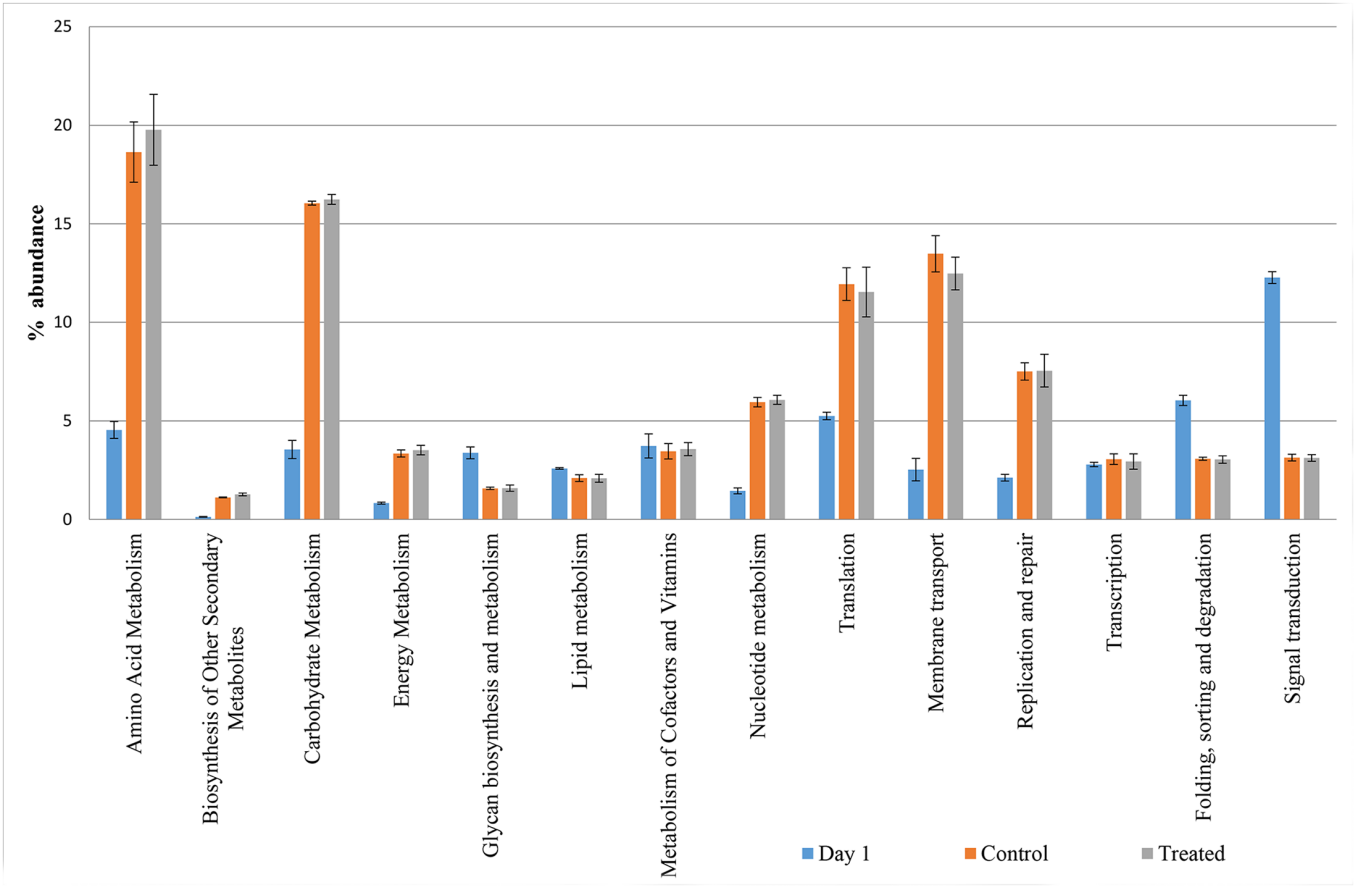
Mean relative frequency of abundance (% abundance) of the KEGG in caeca of day 1 chickens (Day 1) and in ceca of chickens treated with *L*. *acidophilus* (Treated) in comparison to the untreated birds (Control) at 41 days.

The mean relative abundance of the KEGG pathways related to metabolism and genetic information processing in one-day old chicks corresponded to 20.9 and 16.2%, respectively. These values were significantly lower than those detected at 41 days in both CON and LA groups (53.8 vs 55.8% and 25.6 vs 25.0%, respectively) (Fig 4). On the contrary, the environmental information processing and cellular processes pathways were significantly higher in one-day old chicks. In relation to specific metabolism pathways, one-day old chickens showed relative frequencies of abundance of aminoacid and carbohydrate metabolisms significantly lower (4.54 and 3.55%, P<0.001) than those detected in both LA and CON (19.8 and 18.6%; 16.2 and 16.1%, respectively) (Fig 5). The biosynthesis of other secondary metabolites was the only metabolism pathway significantly higher in LA than CON birds (1.27 vs 1.12% respectively; P<0.012) (Fig 5).

**Fig 6:**
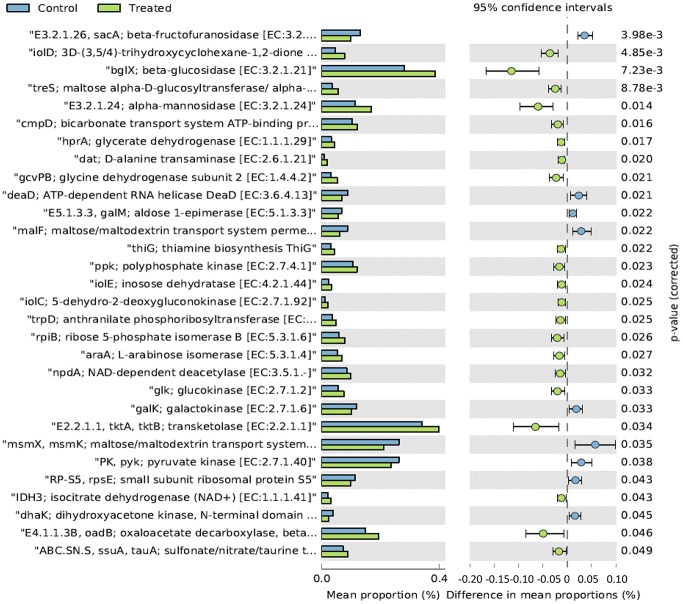
Mean relative frequency of abundance (% abundance) of the KEGG functions showing P < 0.05 between chickens treated with *L*. *acidophilus* (Treated) in comparison to the untreated birds (Control) at 41 days.

**Fig 7:**
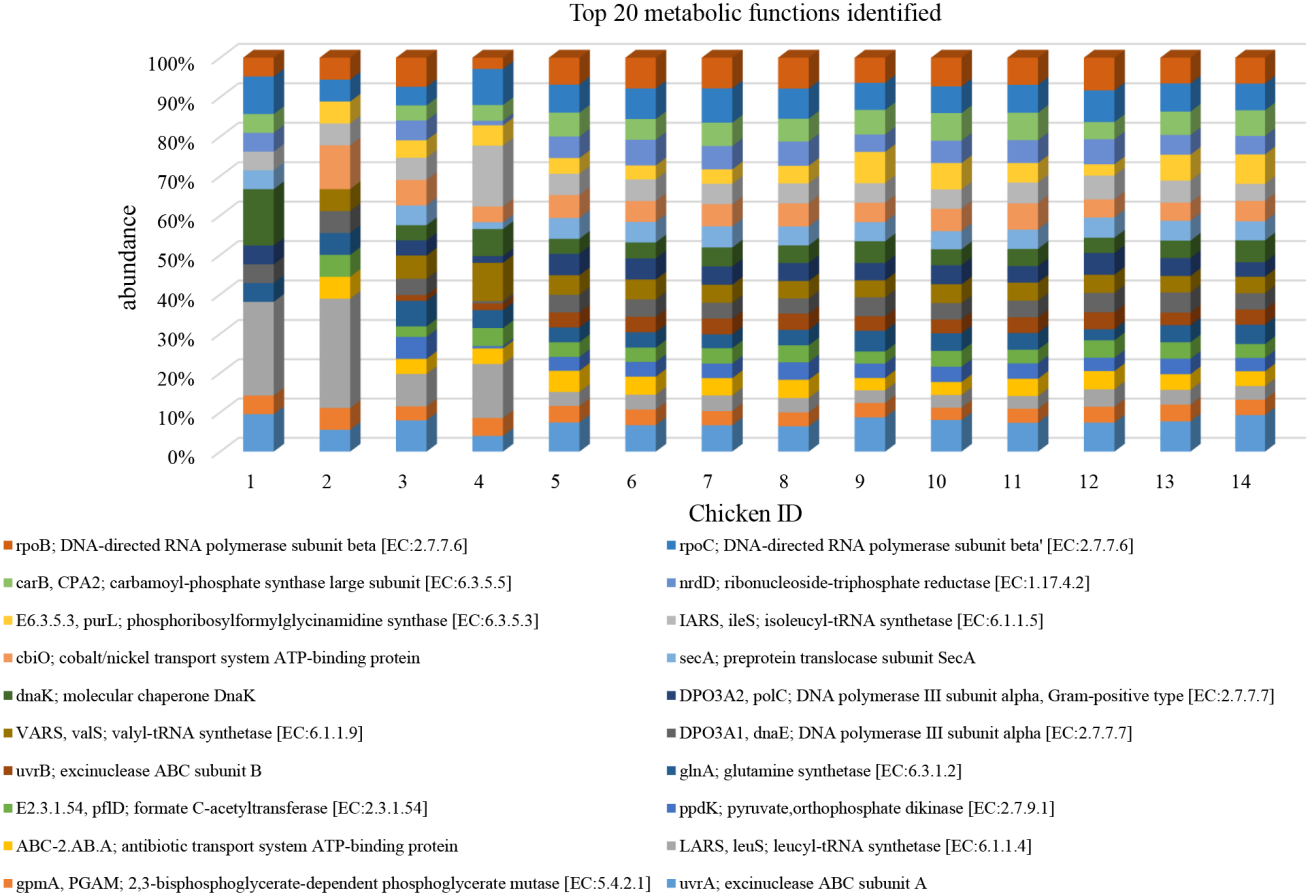
Mean relative frequency of abundance (% abundance) of the top 20 KEGG functions in each of the 14 chickens tested (Day 1: Chicken ID 1–4; Control 41 days: Chicken ID 5–9; Treated 41 days: Chicken ID 6–10).

Overall, in terms of mean relative frequency of abundance, the top 20 metabolic functions identified using the KEGG database are reported in Table 8. At the end of the rearing period the following functions resulted significantly higher in LA in comparison to CON (Fig 6): bglX beta-glucosidase (EC:3.2.1.21), tkatkb transketolase EC:2.2.1.1, alpha-mannosidase (EC:3.2.1.24), ppk polyphosphate kinase (EC:2.7.4.1), oadB oxaloacetate decarboxylase beta subunit (EC: 4.1.1.3), glk glucokinase (EC:2.7.1.2), rpiB ribose 5-phosphate isomerase B (EC: 5.3.1.6), araA L.arabinose isomerase (EC:5.3.1.4) and npdA NAD-dependent deacetylase (EC:3.5.1.-). On the contrary sacA beta-fructofuranosidase (EC:3.3.1.26), malF:maltose/maltodestrin transport system permease, msmX, msmK; maltose/maltodestrine transport system ATP-binding protein, pyk; pyruvate kinase (EC:2.7.1.40) were higher in CON in comparison to LA (Fig 6). The distribution of the top 20 metabolic functions among the 14 tested chickens showed slight differences only in one-day old chicks (Fig 7).

**Table 8:** Mean relative frequency of abundance (%) of the top 20 metabolic functions identified in caeca of one-day old and 41-day old chickens untreated (CON) and treated with *L*. *acidophilus* (LA) by using KEGG database.

Metabolic functions	One-day		CON		LA		CON *vs* LA	One-day *vs* CON	One-day *vs* LA
	Mean	SD	Mean	SD	Mean	SD	P value		
uvrA; excinuclease ABC subunit A	0.06	0.020	0.95	0.09	1.06	0.08	0.129	<0.0001	<0.0001
gpmA. PGAM; 2.3-bisphosphoglycerate-dependent phosphoglycerate mutase [EC:5.4.2.1]	0.04	0.010	0.49	0.03	0.48	0.06	0.799	<0.0001	<0.0001
LARS. leuS; leucyl-tRNA synthetase [EC:6.1.1.4]	0.10	0.020	0.48	0.05	0.48	0.09	0.967	<0.0001	<0.0001
ABC-2.AB.A; antibiotic transport system ATP-binding protein	0.03	0.010	0.54	0.08	0.49	0.06	0.418	0.0002	0.0001
ppdK; pyruvate.orthophosphate dikinase [EC:2.7.9.1]	0.03	0.007	0.50	0.04	0.49	0.06	0.910	<0.0001	<0.0001
E2.3.1.54. pflD; formate C-acetyltransferase [EC:2.3.1.54]	0.03	0.007	0.47	0.05	0.50	0.08	0.632	<0.0001	0.0003
glnA; glutamine synthetase [EC:6.3.1.2]	0.06	0.020	0.51	0.03	0.53	0.06	0.492	<0.0001	<0.0001
uvrB; excinuclease ABC subunit B	0.02	0.010	0.52	0.03	0.53	0.06	0.631	<0.0001	<0.0001
DPO3A1. dnaE; DNA polymerase III subunit alpha [EC:2.7.7.7]	0.03	0.020	0.53	0.02	0.56	0.06	0.371	<0.0001	<0.0001
VARS. valS; valyl-tRNA synthetase [EC:6.1.1.9]	0.05	0.009	0.58	0.04	0.58	0.06	0.860	<0.0001	<0.0001
DPO3A2. polC; DNA polymerase III subunit alpha. Gram-positive type [EC:2.7.7.7]	0.03	0.010	0.60	0.07	0.58	0.13	0.880	<0.0001	0.001
dnaK; molecular chaperone DnaK	0.06	0.020	0.62	0.04	0.64	0.05	0.615	<0.0001	<0.0001
secA; preprotein translocase subunit SecA	0.03	0.010	0.66	0.05	0.64	0.06	0.620	<0.0001	<0.0001
cbiO; cobalt/nickel transport system ATP-binding protein	0.07	0.030	0.70	0.09	0.65	0.08	0.499	<0.0001	<0.0001
IARS. ileS; isoleucyl-tRNA synthetase [EC:6.1.1.5]	0.07	0.010	0.66	0.02	0.66	0.11	0.946	<0.0001	0.0004
E6.3.5.3. purL; phosphoribosylformylglycinamidine synthase [EC:6.3.5.3]	0.03	0.010	0.58	0.18	0.69	0.15	0.407	0.0039	0.0009
nrdD; ribonucleoside-triphosphate reductase [EC:1.17.4.2]	0.04	0.020	0.74	0.10	0.74	0.12	0.994	<0.0001	0.0002
carB. CPA2; carbamoyl-phosphate synthase large subunit [EC:6.3.5.5]	0.04	0.010	0.78	0.03	0.82	0.09	0.453	<0.0001	<0.0001
rpoC; DNA-directed RNA polymerase subunit beta' [EC:2.7.7.6]	0.05	0.002	1.03	0.13	0.95	0.14	0.450	0.0001	0.0002
rpoB; DNA-directed RNA polymerase subunit beta [EC:2.7.7.6]	0.07	0.030	1.00	0.09	0.96	0.15	0.711	<0.0001	0.0002

## Discussion

The administration of probiotic *Lactobacillus* has been demonstrated to stimulate immune responses [6, 33] and improve digestive health [34], as well as growth performance [35–37] in poultry. *Lactobacillus* administration has also been shown to reduce colonization by *Campylobacter* [38, 39], *Clostridium* [40], and *Salmonella* [38, 41], improving the microbial food safety of poultry meat. The ability of *Lactobacillus* species to adhere to epithelial tissues and colonize poultry gut has been reported [42–44]. However, the most important microbial factors implicated in *Lactobacillus* GI persistence in poultry are not well characterized. The lack of a species-specific cell culture model prevented investigations of *Lactobacillus* adhesion and its contribution to GI colonization.

In this study, the supplementation with *L*. *acidophilus* to growing chicken diet produced a significant beneficial effect, with particular regard to body weight gain up to 28 days of age (1,531 vs 1,498 g, respectively for LA and CON; P<0.05) and feed efficiency for the overall experimental period (1.588 vs 1.613 respectively for LA and CON; P<0.05). According to other authors [45–47], the results of this study show that the feed supplementation with *Lactobacillus* improves body weight gain and feed efficiency. These results can be attributed to the improvement of the intestinal microbial balance of the host through a competitive exclusion mechanism and antagonism [10]. Indeed it is well documented that the mode of action of *Lactobacillus* consists in competitive exclusion against harmful bacteria in the gut in favor of beneficial microbial populations, leading to improvement in nutrients utilization of feed [10, 46]. After studying the effect of *Lactobacillus* species supplementation in chickens, Award et al. [48] attributed the improvement of growth performance of broiler chickens to the greater development of small intestinal villi, particularly villus height:crypt depth *ratio* in both duodenum and ileum. In this study, treated birds showed a lower incidence of pasty vent, both at 14 and 28 days of age, probably due to both a better utilization of nutrients and prevalence of healthy conditions in the gut. In general, the reduction of pasty vent incidence is observed in birds with improvement nutrients digestibility, particularly crude fat and protein [49].

In line with previous studies [50], Firmicutes and Proteobacteria were the most common phyla identified in caeca tested in our research. At finer taxonomic resolutions scale, the majority of sequences belonged to various members of Clostridia class. In the one-day old chicks the most represented bacteria genera were *Lactobacillus*, *Clostridium*, *Blautia*, *Escherichia*, *Enterococcus*, *Eubacterium* and *Ruminococcus*. This trend was partially observed also in the chickens at 41 days. However, at the end of the rearing period the most representative genera were *Faecalibacterium*, *Subdonigranulum*, *Roseburia* and *Eubacterium*. The presence of *Clostridium*-related species in the chicken caeca was observed by other authors [51–53]. *Clostridium* clusters IV (including *Faecalibacterium prausnitzzi*, *Subdonigranulum variabile* and *Anaerotruncus colihominis*) and XIVa (including *Roseburia intestinalis* and *Ruminococcus torques)* produce primarily butyrate [54]. Among these species, *Faecalibacterium prausnitzzi* and *Subdonigranulum variabile* were not significantly different between LA and CON (Table 7), whereas *Roseburia intestinalis* and *Ruminococcus torques* were significantly higher in LA (Fig 3). Differences in the abundance of *Anaerotruncus colihominis* between the two groups were not analyzed for its low abundance (Table 6). *Faecalibacterium prausnitzzi* has a requirement for acetate, and produces butyrate, formate and lactate [55]. Butyric acid has been shown to have an important function in protection against pathogens in poultry [56]. Furthermore, it is involved in several intestinal functions, being an energy source stimulating the epithelial cells proliferation and differentiation, other than exerting an antimicrobial effect by promoting the production of peptides and stimulating the production of tight junction protein [57]. Overall, the microbiological profiles identified in one-day old chicks, as well as CON and LA, partially confirm those reported by other authors, showing that the first days after hatching broiler caecum is colonized by facultative aerobes bacteria [58]. Oxygen consumption by those bacteria drives the lower gut environment to more reducing conditions, facilitating subsequent growth and colonization by extremely oxygen-sensitive obligate anaerobes [59–61].

The relative abundance of *Lactobacillus acidophilus* in the caeca of LA chickens was comparable with that of CON group. This result might be explained taking into account the colonization preference of the administered strain for the crop and the small intestine, even if this specific aspect was not investigated. Besides the lack of colonization of *Lactobacillus acidophilus* in broiler caeca, the results of this study suggest that the metabolic activity of supplemented *Lactobacillus acidophilus* positively affects the microbial species producing butyric acid by a cross feeding mechanism.

*Ruminococcus torques* was significantly higher in CON group in comparison to LA at 41 days. *R*. *torques* is known to degrade GI mucin [62], representing a carbon and energy source for intestinal microbiota. It has been estimated that 1% of colonic microbiota is able to degrade host mucin using enzymes (e.g. glycosidases and sulfatases) that can degrade the oligosaccharide chains [63, 64]. Moreover, degradation of mucin is regarded as a pathogenicity factor, since loss of the protective mucus layer may expose GI tract cells to pathogens [65]. Therefore, the higher abundance of *R*. *torques* in CON might be possibly related to the higher incidence of pasty vent in CON in comparison to LA group, but this hypothesis needs to be confirmed.

In relation to the metabolic functions, LA group showed a significantly higher level of β-glucosidase (Fig 6). This enzyme contributes to the hydrolysis of glucose monomers from non-starch polysaccharides (e.g., cellulose, β-glucans), playing an important role in the fermentation of undigested carbohydrates and, ultimately, in animal performance and health. In particular, β-glucosidase (β-glucoside glucohydrolase; EC3.2.1.21) hydrolyzes alkyl- and aryl-β-glucosides, as well as diglucosides and oligosaccharides, to release glucose and an aglycone [66]. It also hydrolyzes isoflavonal glycoside conjugates into isoflavone aglycones, such as genistein, daidzein, and glycitein. An increase of the concentrations of genistein and daidzein in soy milk has been reported by using strains of *Streptococcus thermophilus*, *L*. *acidophilus*, *L*. *delbrueckii ssp*. *bulgaricus*, *L*. *casei*, *L*. *plantarum*, *L*. *fermentum* and several *Bifidobacterium* species [67, 68]. These aglycones hydrolyzed by β-glucosidases from intestinal microorganisms are readily absorbed across the villi of the intestine [69], possess greater bioavailability than the corresponding glycoside conjugates [70] and a wide range of biological properties, such as antioxidant and anti-tumor activities [71, 72]. In broilers fed with 0.2% β-glucosidase, a significant increase in average daily weight gain (P<0.05) and feed conversion ratios (P<0.05) were observed in comparison to controls [73].

## Conclusions

The supplementation with *Lactobacillus acidophilus* D2/CSL (CECT 4529) at the recommended dietary dosage of 1x10^9^ cfu/kg feed in broiler chickens significantly improved body weight at 28 days (commercial weight of 1.5 kg) and feed conversion rate from 0 to 41 days. In addition, the incidence of pasty vent was reduced in LA birds. The relative abundance of *Lactobacillus acidophilus* in the caeca of LA chickens was comparable with that of CON group. However, a positive effect of the supplementation with *Lactobacillus acidophilus* was observed in relation to the metabolic functions in the treated group, with particular reference to the higher abundance of β-glucosidase, improving animal performances and health.
